# Highly pathogenic H5N6 influenza A viruses recovered from wild birds in Guangdong, southern China, 2014–2015

**DOI:** 10.1038/srep44410

**Published:** 2017-03-15

**Authors:** Yinfeng Kang, Lu Liu, Minsha Feng, Runyu Yuan, Can Huang, Yangtong Tan, Pei Gao, Dan Xiang, Xiaqiong Zhao, Yanling Li, David M. Irwin, Yongyi Shen, Tao Ren

**Affiliations:** 1 College of Veterinary Medicine, South China Agricultural University, Guangzhou 510642, China; 2 Key Laboratory of Zoonosis Prevention and Control of Guangdong Province, Guangzhou 510642, China; 3 State Key Laboratory of Oncology in South China, Collaborative Innovation Center for Cancer Medicine, Department of Experimental Research, Sun Yat-sen University Cancer Center, Guangzhou, 510060, China; 4 Shantou University Medical College, Shantou 515041, China; 5 Key Laboratory for Repository and Application of Pathogenic Microbiology, Research Center for Pathogens Detection Technology of Emerging Infectious Diseases, Guangdong Provincial Center for Disease Control and Prevention, Guangzhou 510000, China; 6 Department of Laboratory Medicine and Pathobiology, University of Toronto, Toronto, M5S 1A8, Canada; 7 Banting and Best Diabetes Centre, University of Toronto, Toronto, M5S 1A8, Canada

## Abstract

Since 2013, highly pathogenic (HP) H5N6 influenza A viruses (IAVs) have emerged in poultry in Asia, especially Southeast Asia. These viruses have also caused sporadic infections in humans within the same geographic areas. Active IAV surveillance in wild birds sampled in Guangdong province, China from August 2014 through February 2015 resulted in the recovery of three H5N6 IAVs. These H5N6 IAV isolates possess the basic amino acid motif at the HA1-HA2 cleavage site that is associated with highly pathogenic IAVs infecting chickens. Noteworthy findings include: (1) the HP H5N6 IAV isolates were recovered from three species of apparently healthy wild birds (most other isolates of HP H5N6 IAV in Asia are recovered from dead wild birds or fecal samples in the environment) and (2) these isolates were apparently the first recoveries of HP H5N6 IAV for two of the three species thus expanding the demonstrated natural host range for these lineages of virus. This investigation provides additional insight into the natural history of HP H5N6 IAVs and identifies the occurrence of non-lethal, HP H5N6 IAV infections in wild birds thereby demonstrating the value of active IAV surveillance in wild birds.

Avian influenza A viruses (AIVs) pose significant threats to animal and human health. Among the 18 influenza haemagglutinin (HA) subtypes, the H5 subtype, in combination with a variety of individual neuraminidase (NA) subtypes (Nx), cause the most frequent and widespread epizootics resulting in severe economic losses to the poultry industry^1^,^2^. In addition several H5Nx influenza A virus (IAV) combinations have also been proven to be zoonotic, thereby raising global concern for human health. The most recent emergent lineages of concern are the highly pathogenic (HP) and/or zoonotic lineages of H5N6 AIVs in China^3^,^4^,^5^,^6^ and Southeast Asia^7^, and more recently in Korea^8^. These viruses have caused human infections in China since 2014^9^. Human infections of H5N6 viruses reappeared in December 2015, in Guangdong province, China^10^. This has raised concerns about the threat of these viruses to public health.

Poultry are usually considered to be the source of the human infections of AIVs, and a lot of work has focused on live poultry markets^10^. Wild birds are considered to be natural reservoirs for lowly pathogenic AIVs as well as being an important source of low pathogenic AIVs infecting poultry^11^. The Qinghai-like highly pathogenic avian influenza A (HPAI) H5N1 viruses caused epizootic outbreaks in wild birds in 2005, and were rapidly disseminated throughout Africa, Europe and the Middle East along flyways by migratory birds resulting in hundreds of cases of human infection and the death of tens of millions of poultry^12^. Also, H5N8 HPAIVs appeared to be spread by long-distance migratory birds^13^ which add additional evidence supporting the role of wild birds in the long-distance transmission and spread of HPAIVs during their seasonal migrations along their flyways^14^. In this investigation we examined wild birds in Guangdong Province for active IAV infections from August 2014 through February 2015.

## Result

### Influenza virus surveillance in wild birds

From August 2014 to February 2015, a total of 1092 oropharyngeal and cloacal samples were collected from apparently healthy wild birds in Guangdong Province, China (Table 1). 11 HA-positive specimens were recovered. Three H5N6 subtype AIVs were isolated from an oriental magpie-robin (oropharyngeal swab), a common moorhen (oropharyngeal swab), and a Pallas’s sandgrouse (both oropharyngeal and cloacal swabs). These H5N6 AIVs were designated as A/oriental magpie-robin/Guangdong/SW8/2014 (H5N6) (SW8), A/common moorhen/Guangdong/GZ174/2014 (H5N6) (GZ174) and A/Pallas’s sandgrouse/Guangdong/ZH283/2015 (H5N6) (ZH283) respectively.

### Phylogenetic analyses of the H5N6 viruses

**Figure 1:**
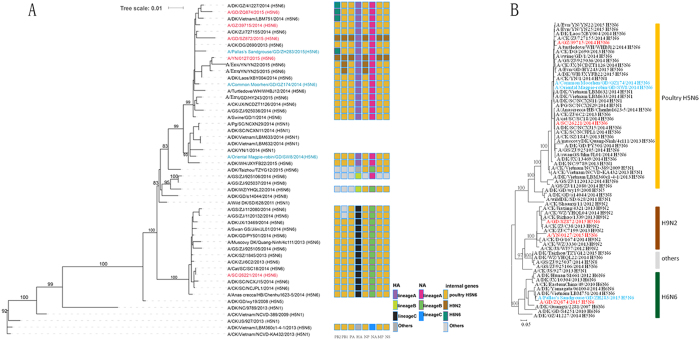
Phylogenetic analyses of complete hemagglutinin (**A**) and PB2 (**B**) genes of indicated H5N6 viruses. Viruses highlighted in blue were characterized in this study. Human-isolated H5N6 are marked in red. Genotypes of the influenza viruses are shown on the right (**A**) as eight coloured blocks representing each gene segment (from left to right: PB2, PB1, polymerase acidic, haemagglutinin, nucleoprotein, neuraminidase, matrix and non-structural) with the colour indicating the lineage of that segment. Host species are: CK (chicken), DK (duck), GS (goose), PG (pigeon). Geographic locations are: ZJ (Zhejiang), GD (Guangdong), JX (Jiangxi), YN (Yunnan), DG (Dongguan), JS (Jiangsu), GZ (Guangzhou), Env (environment), SC (Sichuan), WZ (Wenzhou), SD (Shandong), SZ (Shenzhen), NC (Nanchang), WH (Wuhan), and HB (Hubei).

We constructed maximum likelihood trees for each of the eight viral genomic segments from our new sequences combined with data from public sources (listed in Supplementary Table 1). The HA gene of our three new isolates grouped with clade 2.3.4.4 H5 of influenza viruses (Fig. 1A). Phylogenetic analysis of the N6-NA gene showed that it could be delineated into two major lineages (marked as lineages A and B, in Supplementary Figure 1A).

Except for PB2, the other genomic segments (HA, NA, PB1, PA, NP, MP and NS) of these three IAV isolates cluster with poultry H5N6 viruses (Supplementary Figure 1B–F). The gene sequences of ZH283 (HA, 99.2%; NA, 98.9%; PB1, 99.3%; PA, 99.3%; NP, 99.7%; MP, 99.3%; and NS, 99.2%), SW8 (HA, 98.4%; NA, 99.7%; PB1, 99.1%; PA, 99.3%; NP, 99.3%; MP, 99.3%; and NS, 99.1%), and GZ174 (HA, 99.7%; NA, 99.9%; PB1, 99.2%; PA, 99.1%; NP, 99.5%; MP, 98.9%; and NS, 99.0%) have high identity with those of A/chicken/Dongguan/2690/2013 (H5N6). For PB2, GZ174 and SW8 cluster with poultry H5N6 viruses, and have high identify with that of A/chicken/Dongguan/2690/2013 (H5N6) (99.4%, and 99.4%, respectively). While the PB2 gene of ZH283 is derived from poultry H6N6 (Fig. 1B), and has the highest identify (98.3%) with that of A/duck/Yamagata/061004/2014 (H6N6).

### Molecular characterization

The SW8, GZ174 and ZH283 IAV isolates have a string of basic amino acids at the HA cleavage site (-RRRKR↓G-), which is characteristic of HPAIVs^15^,^16^. The HA1 receptor-binding pocket of all three isolates retained the amino acid residues Q226 and G228 (H3 numbering), which preferentially recognize the avian influenza virus receptor (α-2,3 galactose sialic acids) (Supplementary Table 2)^17^. However, positions 138 A and 155 T of these three isolates (except position I155 of the ZH283 virus, which was not mutated) indicate that these isolates should also have affinity for human-type receptors (α-2,6 galactose sialic acids)^18^,^19^ (Supplementary Table 2).

The three isolates have an eleven amino acid residue deletion (-TIINNHPQNNF-) in the stalk of the NA protein, which contributes to increased virulence in mice^20^. The H274Y mutation was not found in the NA protein, indicating that these isolates might be sensitive to neuraminidase inhibitor drugs such as Oseltamivir (Tamiflu)^21^,^22^.

The PB2 genes of these three isolates do not have the mutations E627K or D701N, which are considered to be the predominant factor for cross-species transmission from avian to mammals^23^,^24^. The amino-acid substitution S31N in the M2 protein was not observed, indicating that the three isolates should be sensitive to influenza A M2 protein inhibitors such as amantadine^25^. Studies have shown that the virulence of the influenza virus in humans is associated with viral resistance to the antiviral effects of cytokines, such as interferons, and that the mutation D92E in the NS1 protein promotes greater resistance to these cytokines^26^, and all three isolates characterized in this study had the D92E mutation in the NS1 protein (Supplementary Table 2).

## Discussion

From August 2014 through February 2015, our active surveillance efforts resulted in the recovery of three H5N6 (SW8, GZ174 and ZH283) IAV isolates from wild birds (Table 1). The sequences of these isolates have a string of basic amino acids at the HA cleavage site (-RRRKR↓G-), which is characteristic of HPAIVs^15^,^16^. Our study supports that Palla’s sandgrouse (ZH283) and the common moorhen (GZ174) are within the host range of HP H5N6 AIVs currently circulating in poultry and sporadically infecting humans in Asia. These wild birds do not migrate very long-distance. However, H5N6 IAVs can infect wild birds, such as swan geese^27^ and teal ducks^28^, which are long-distance migrators. The common moorhen, swan geese and teal ducks have overlapping habitats across the Eurasia (data from Avibase database: https://avibase.bsc-eoc.org). Wild birds have a proven role in the long-distance spread of HPAIVs, such as H5N1 and H5N8^11^,^12^,^13^ along their migratory flyways. The detection of H5N6 AIVs in apparently healthy wild birds in this study raises the possibility that wild birds might carry H5N6 to other areas.

The gene sequences of these three H5N6 isolates shared high identity for all genes except PB2. The PB2 genes of SW8 and GZ174 have high identify with those of poultry H5N6 viruses (such as A/chicken/Dongguan/2690/2013 (H5N6)). While the PB2 of ZH283 clustered with A/duck/GZ/41227/2014 (H5N6), A/Guangdong/ZQ874/2015 (H5N6), and A/duck/Vietnam/LBM751/2014 (H5N6), and is derived from duck H6N6 (Fig. 1B). The H5N6 AIVs have different reassortments. Although sequence analysis suggest that these viruses preferentially recognize the avian influenza virus receptor (α-2,3 galactose sialic acids), they have some amino acids that are associated with affinity for human-type receptors (α-2,6 galactose sialic acids). Also these three isolates all have an eleven amino acid residue deletion in the stalk of the NA protein, which is associated with increased virulence in mice^20^. Thus, it’s possible that they could pose a threat to public health.

The H5N6 viruses have circulated within poultry in China and have caused several human infection cases since 2013^3^,^4^,^5^,^10^. In this study, we systematically analyzed the genetics and phylogeny of three HPAV H5N6 isolates obtained from wild birds in southern China. We found that the H5N6 HPAIVs had different reassortments and affected several species of wild birds in southern China. Future large-scale surveillance efforts of wild birds and poultry are needed to reveal the circulation of these HPAIVs.

## Materials and Methods

### Ethics Statement

We conducted all animal experiments under the guidance of both the Guangdong Provincial Center for Disease Control and Prevention’s Institutional Animal Care and Use Committee and the Association for Assessment and Accreditation of Laboratory Animal Care International. South China Agricultural University’s Committee on the Ethics of Animal Experiments of Animal Biosafety Level 3 reviewed and approved the study protocols (permit no. 2015–10). The methods were carried out in accordance with these approved guidelines.

### Sample Collection

Oropharyngeal and cloacal swab samples were collected from wild birds from August 2014 through February 2015 in Guangdong province, southern China (Table 1). Wetlands or lakes of three cities (Guangzhou, Zhuhai and Shanwei) in Guangdong province were chosen for this surveillance. These three cities represent the three regions of the Pearl River Delta Region. Mist nets and traps were used to capture wild birds. The Forest Police Administration provided some of the samples (e.g. Chinese hwamei and Pallas’s sandgrouse). Oropharyngeal and cloacal swab samples were taken and placed separately in 1.0 ml of transport medium (medium 199) containing antibiotics, and kept in cool boxes until they arrived in the laboratory.

### Virus isolation, identification and genomic sequencing

The protocols for virus isolation, and identification were described in our previous studies^29^. Briefly, samples were inoculated into 9–11 day old embryonated chicken eggs for 48 to 96 hours at 37 °C. Three eggs were inoculated per sample. Allantoic fluid was harvested and tested for the presence of active hemagglutinin. Haemagglutinin-positive isolates were further tested by real-time RT-PCR for the influenza matrix gene, and subtyped by the haemagglutination and neuraminidase inhibition (HAI and NAI) assays.

All three H5N6 positive samples of this surveillance (Table 1) were chosen for viral genome sequencing. Firstly, viral RNA was extracted from the first passage allantoic fluid from the eggs using the QIAamp Viral RNA Minikit (Qiagen, Germany). PCR amplification was performed using segment-specific primers as previously described^30^. PCR products were purified with the QIAquick PCR purification kit (QIAGEN) and sequenced using an automatic ABI Prism 3730 genetic analyzer (Applied Biosystems). The full length genomic sequences of the three H5N6 IAV isolates, A/Oriental Magpie-robin/Guangdong/SW8/2014 (H5N6), A/Common Moorhen/Guangdong/GZ174/2014 (H5N6) and A/Pallas’s Sandgrouse/Guangdong/ZH283/2015 (H5N6), were deposited into GenBank (accession numbers: KT454936-KT454959).

### Phylogenetic analyses

On 20 January 2016, all available H5, H6N6 and H9N2 AIV subtype sequences were collated from GenBank and the Global Initiative on Sharing Avian Influenza Data (GISAID). These sequences were combined with the genome sequences obtained in this study, and the sequences of each gene were separately aligned using the European Bioinformatics Institute’s MUSCLE^31^. Phylogenetic trees were constructed using RAxML v8.2.4^32^, and 66 sequences that appeared to be the most closely related to the H5N6 outbreak isolates were selected for further analysis (listed in Supplementary Table 1). Maximum likelihood phylogenies of these sequences were constructed using PhyML with bootstrap analysis (1,000 replicates)^33^. The phylogenies deduced for all segments are shown in Fig. 1 and Supplementary Figure 1.

## Additional Information

**How to cite this article:** Kang, Y. *et al*. Highly pathogenic H5N6 influenza A viruses recovered from wild birds in Guangdong, southern China, 2014–2015. *Sci. Rep.* **7**, 44410; doi: 10.1038/srep44410 (2017).

**Publisher's note:** Springer Nature remains neutral with regard to jurisdictional claims in published maps and institutional affiliations.

## Supplementary Material

Supplementary Materials

## Figures and Tables

**Table 1:** Surveillance statistics for Influenza A virus in Guangdong during 2014–2015.

City	Sampling time	Site	Common name	Scientific name	No. of sample	No. of positive	Positive rate (%)	Subtype (n)^a^
Guangzhou	08-9-2014	wetland	common moorhen	*Gallinula chloropus*	18	1	5.56	H5N6 (1)
			great white egret	*Ardea alba*	6	0	0	
			little egret	*Egretta garzetta*	8	0	0	
			night heron	*Nycticorax nycticorax*	8	0	0	
			gray heron	*Ardea cinerea*	14	0	0	
			cattle egret	*Bubulcus ibis*	10	0	0	
			Chinese pond heron	*Ardeola bacchus*	11	0	0	
			rose-ringed parrakeet	*Psittacula krameri*	13	0	0	
		Subtotal			88	1	1.14	
	16-9-2014	lake	night heron	*Nycticorax nycticorax*	18	0	0	
			common kingfisher	*Alcedo atthis*	16	0	0	
			white-cheeked starling	*Spodiopsar cineraceus*	24	3	12.5	H5N1 (3)
			sooty-headed bulbul	*Pycnonotus aurigaster*	4	0	0	
			little egret	*Egretta garzetta*	35	0	0	
			cormorant	*Phalacrocorax carbo*	3	0	0	
			heuglin’s gull	*Larus heuglini*	8	0	0	
			purple heron	*Ardea purpurea*	14	0	0	
			gray heron	*Ardea cinerea*	20	0	0	
		Subtotal			142	3	2.11	
Zhuhai	2- 9-2014	wetland	common moorhen	*Gallinula chloropus*	20	0	0	
			common gull	*Larus canus*	46	0	0	
			little egret	*Egretta garzetta*	14	0	0	
			night heron	*Nycticorax nycticorax*	28	0	0	
			Chinese hwamei	*Garrulax canorus*	6	1	16.7	H5N1 (1)
			Eurasian Tree sparrow	*Passer montanus*	4	0	0	
			white-breasted waterhen	*Amaurornis phoenicurus*	24	0	0	
			vega gull	*Larus vegae*	36	0	0	
		Subtotal			178	1	0.56	
	20-2-2015	wetland	night heron	*Nycticorax nycticorax*	36	0	0	
			common moorhen	*Gallinula chloropus*	120	0	0	
			common gull	*Larus canus*	58	0	0	
			Pallas's sandgrouse	*Syrrhaptes paradoxus*	42	1	2.38	H5N6 (1)
			purple heron	*Ardea purpurea*	42	0	0	
			little egret	*Egretta garzetta*	36	0	0	
			whooper swan	*Cygnus cygnus*	6	0	0	
			daurian redstart	*Phoenicurus auroreus*	4	0	0	
			plain prinia	*Prinia inornata*	12	0	0	
			dusky warbler	*Phylloscopus fuscatus*	11	0	0	
			Japanese white-eye	*Zosterops japonicus*	4	0	0	
			green-winged teal	*Anas crecca*	8	0	0	
			spot-billed duck	*Anas poecilorhyncha*	12	2	16.67	H6N6 (2)
			common kingfisher	*Alcedo atthis*	14	0	0	
		Subtotal			405	3	0.74	
Shanwei	02-8-2014	wetland	rose-ringed parrakeet	*Psittacula krameri*	10	0	0	
			night heron	*Nycticorax nycticorax*	20	0	0	
			oriental magpie-robin	*Copsychus saularis*	14	1	7.14	H5N6 (1)
			common moorhen	*Gallinula chloropus*	12	0	0	
			great white egret	*Ardea alba*	6	0	0	
			little egret	*Egretta garzetta*	8	0	0	
			purple heron	Ardeapurpurea	9	0	0	
			gray heron	Ardeacinerea	5	0	0	
		Subtotal			84	1	1.19	
	25-12-2014	lake	common moorhen	*Gallinula chloropus*	14	0	0	
			spotted dove	Streptopeliachinensis	18	0	0	
			night heron	*Nycticorax nycticorax*	20	0	0	
			purple heron	*Ardea purpurea*	8	0	0	
			great white egret	*Ardea alba*	13	0	0	
			swan goose	*Anser cygnoides*	31	2	6.45	H5N1 (2)
			daurian redstart	*Phoenicurus auroreus*	4	0	0	
			dusky warbler	*Phylloscopus fuscatus*	12	0	0	
			grebe	Podicipediformes	6	0	0	
			black-throated stone-chat	*Saxicola torquata*	4	0	0	
			spot-billed duck	*Anas poecilorhyncha*	22	0	0	
			turtle dove	*Streptopelia orientalis*	43	0	0	
		Subtotal			195	2	1.03	
		Total			1092	11	1.01	

Note: ^a^“n” represents the number of positive samples.
