# Supplementary material for: Highly pathogenic H5N6 influenza A viruses recovered from wild birds in Guangdong, southern China, 2014–2015
Source: Sci Rep. 2017 Mar 15;7:44410. doi: 10.1038/srep44410 (PMC5353559; doi:10.1038/srep44410)
Supplement: Supplementary Materials [file srep44410-s1.doc]

**Highly pathogenic H5N6 influenza A viruses recovered from wild birds in Guangdong, southern China, 2014-2015**

Yinfeng Kang^a,b,g^, Lu Liu^a,c^, Minsha Feng^a,b^, Runyu Yuan^a,b,d^, Can Huang^a,c^, Yangtong Tan^a,b^, Pei Gao^a,b^, Dan Xiang^a,c^, Xiaqiong Zhao^a,b^, Yanling Li^a,b^, David M. Irwin^e,f^, Yongyi Shen^a.b*^, Tao Ren^a,b*^

**Supplementary Figure and Tables：**

Supplementary Figure 1. Phylogenetic analyses of six genes of the indicated H5N6 viruses：A) NA; B) PB1; C) PA; D) NP; E) M; F) NS. Viruses highlighted in blue were characterized in this study. Host species are: Ck (chicken), Dk (duck), GS (goose), Pg (pigeon). Geographic locations are: ZJ (Zhejiang), GD (Guangdong), JX (Jiangxi), YN (Yunnan), DG (Dongguan), JS (Jiangsu), GZ (Guangzhou), Evn (environment), SC (Sichuan), WZ (Wenzhou), SD (Shandong), SZ (Shenzhen), NC (Nanchang), WH (Wuhan), and HB (Hubei).


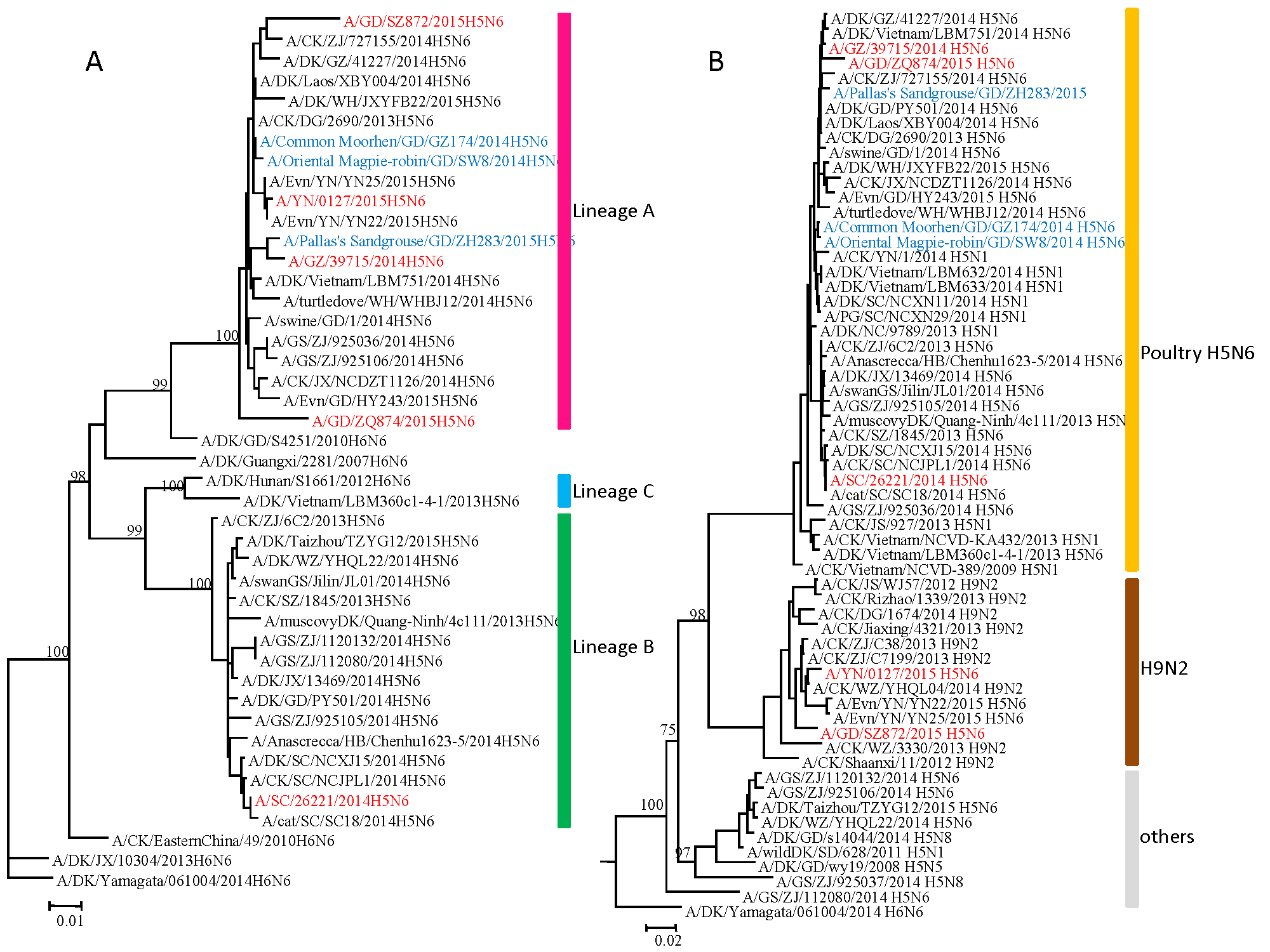


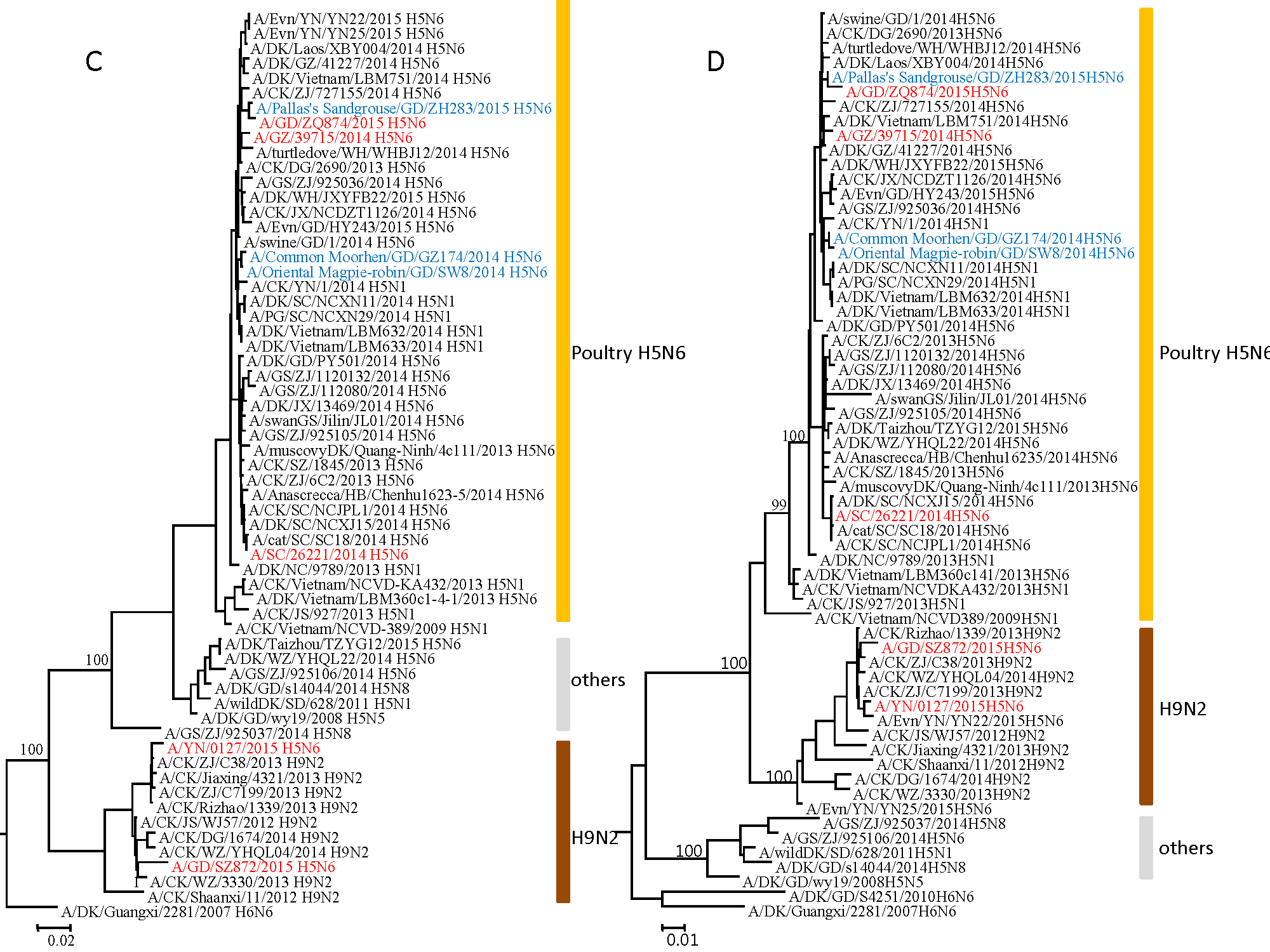


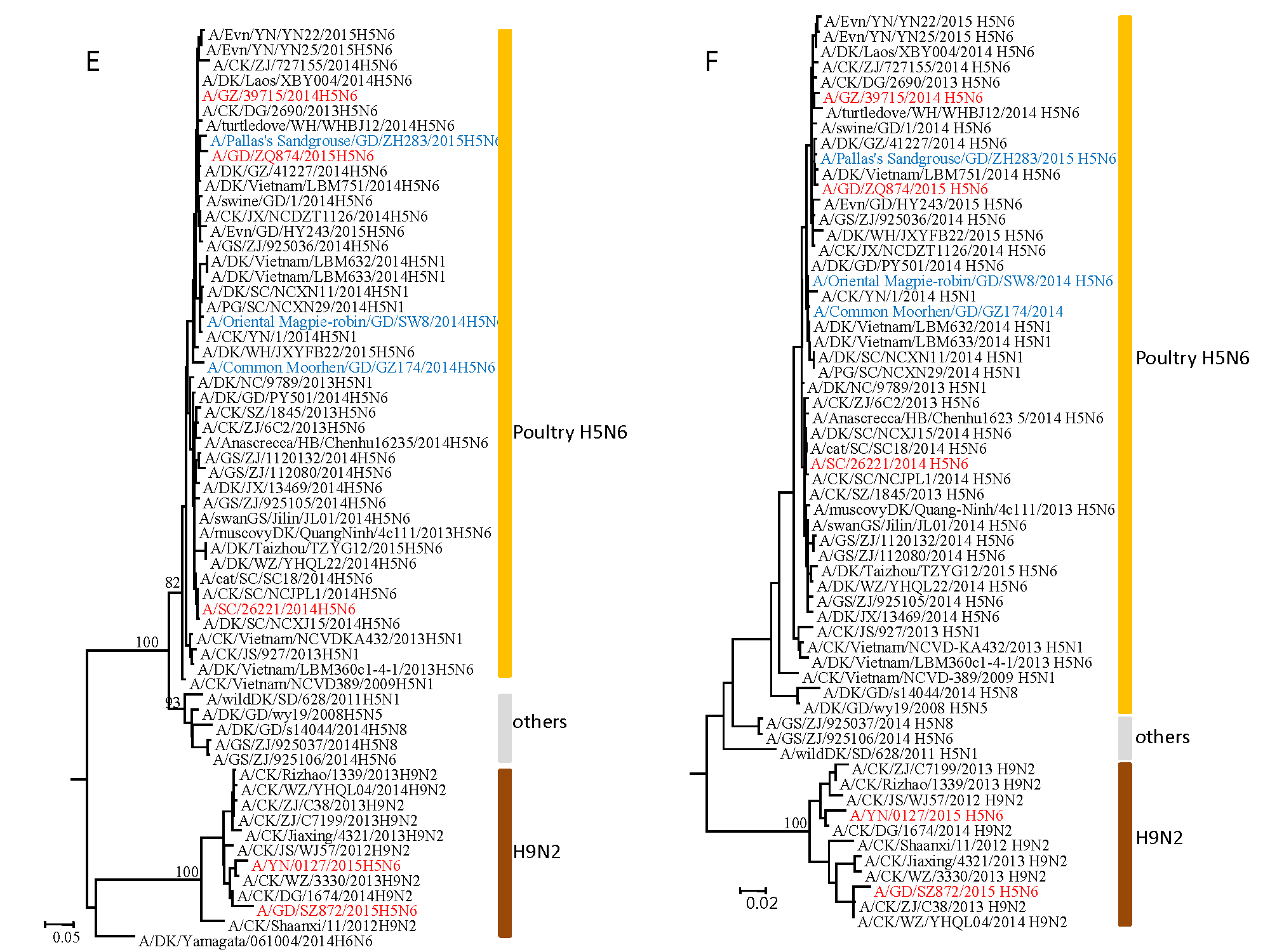


**Supplementary Table 1.** Complete genomes of 66 avian influenza viruses used in this study.

| **Strain name** | **PB2** | **PB1** | **PA** | **HA** | **NP** | **NA** | **M** | **NS** |
| --- | --- | --- | --- | --- | --- | --- | --- | --- |
| A/Anas_crecca/Hubei/Chenhu1623-5/2014(H5N6) | KM251532 | KM251522 | KM251512 | KM251462 | KM251492 | KM251485 | KM251472 | KM251502 |
| A/Guangzhou/39715/2014(H5N6) | KP765785 | KP765786 | KP765787 | KP765788 | KP765789 | KP765790 | KP765791 | KP765792 |
| A/Yunnan/0127/2015(H5N6) | KT245150 | KT245149 | KT245148 | KT245143 | KT245146 | KT245145 | KT245144 | KT245147 |
| A/cat/Sichuan/SC18/2014(H5N6) | KM873635 | KM873636 | KM873637 | KM873638 | KM873639 | KM873640 | KM873641 | KM873642 |
| A/chicken/Dongguan/1674/2014(H9N2) | KP416443 | KP416444 | KP416445 | KP416446 | KP416447 | KP416448 | KP416449 | KP416450 |
| A/chicken/Dongguan/2690/2013(H5N6) | KP286098 | KP286099 | KP286100 | KP286101 | KP286102 | KP286103 | KP286104 | KP286105 |
| A/chicken/Eastern_China/49/2010(H6N6) | JF965053 | JF965092 | JF965103 | JF965143 | JF965182 | JF965320 | JF965223 | JF965295 |
| A/chicken/Jiangsu/927/2013(H5N1) | KP762508 | KP762507 | KP762506 | KP762501 | KP762504 | KP762502 | KP762503 | KP762505 |
| A/chicken/Jiangsu/WJ57/2012(H9N2) | KP893703 | KP893704 | KJ000709 | KJ000710 | KP893705 | KP893706 | KP893707 | KP893708 |
| A/chicken/Jiangxi/NCDZT1126/2014(H5N6) | KP090444 | KP090445 | KP090446 | KP090447 | KP090448 | KP090449 | KP090450 | KP090451 |
| A/chicken/Jiaxing/4321/2013(H9N2) | KP414715 | KP414716 | KP414717 | KP414718 | KP414719 | KP414720 | KP414721 | KP414722 |
| A/chicken/Rizhao/1339/2013(H9N2) | KF260950 | KF260706 | KF260462 | KF259175 | KF259974 | KF259578 | KF259455 | KF260218 |
| A/chicken/Shaanxi/11/2012(H9N2) | KC767264 | KC767257 | KC767258 | KC767259 | KC767260 | KC767261 | KC767262 | KC767263 |
| A/chicken/Shenzhen/1845/2013(H5N6) | KP284978 | KP284979 | KP284980 | KP284981 | KP284982 | KP284983 | KP284984 | KP284985 |
| A/chicken/Sichuan/NCJPL1/2014(H5N6) | KM251533 | KM251523 | KM251513 | KM251463 | KM251493 | KM251486 | KM251473 | KM251503 |
| A/chicken/Vietnam/NCVD_KA432/2013(H5N1) | KP097841 | KP097862 | KP097883 | KP097918 | KP097939 | KP097974 | KP097995 | KP098016 |
| A/chicken/Wenzhou/3330/2013(H9N2) | KP415371 | KP415372 | KP415373 | KP415374 | KP415375 | KP415376 | KP415377 | KP415378 |
| A/chicken/Wenzhou/YHQL04/2014(H9N2) | KU143582 | KU143539 | KU143496 | KU143285 | KU143410 | KU143349 | KU143324 | KU143453 |
| A/chicken/Yunnan/1/2014(H5N1) | KJ683885 | KJ683886 | KJ683887 | KJ683888 | KJ683889 | KJ683890 | KJ683891 | KJ683892 |
| A/chicken/Zhejiang/6C2/2013(H5N6) | KJ807774 | KJ807775 | KJ807777 | KJ807779 | KJ807781 | KJ807783 | KJ807785 | KJ807787 |
| A/chicken/Zhejiang/727155/2014(H5N6) | KU042679 | KU042708 | KU042737 | KU042766 | KU042795 | KU042824 | KU042853 | KU042882 |
| A/chicken/Zhejiang/C38/2013(H9N2) | KU042122 | KU042175 | KU042228 | KU042281 | KU042334 | KU042387 | KU042440 | KU042493 |
| A/chicken/Zhejiang/C7199/2013(H9N2) | KU042111 | KU042164 | KU042217 | KU042270 | KU042323 | KU042376 | KU042429 | KU042482 |
| A/duck/Guangdong/s14044/2014(H5N8) | KT383483 | KT383484 | KT383485 | KT383486 | KT383487 | KT383488 | KT383489 | KT383490 |
| A/duck/Guangdong/wy19/2008(H5N5) | CY091632 | CY091633 | CY091634 | CY091635 | CY091636 | CY091637 | CY091638 | CY091639 |
| A/duck/Guangxi/2281/2007(H6N6) | CY109623 | CY109624 | CY109625 | CY109626 | CY109627 | CY109628 | CY109629 | CY109630 |
| A/duck/Guangzhou/41227/2014(H5N6) | KP765793 | KP765794 | KP765795 | KP765796 | KP765797 | KP765798 | KP765799 | KP765800 |
| A/duck/Hunan/S1661/2012(H6N6) | CY146617 | CY146618 | CY146619 | CY146620 | CY146621 | CY146622 | CY146623 | CY146624 |
| A/duck/Jiangxi/10304/2013(H6N6) | KP285306 | KP285307 | KP285308 | KP285309 | KP285310 | KP285311 | KP285312 | KP285313 |
| A/duck/Jiangxi/13469/2014(H5N6) | KP286418 | KP286419 | KP286420 | KP286421 | KP286422 | KP286423 | KP286424 | KP286425 |
| A/duck/Laos/XBY004/2014(H5N6) | KM496974 | KM496975 | KM496976 | KM496977 | KM496978 | KM496983 | KM496980 | KM496981 |
| A/duck/Nanchang/9789/2013(H5N1) | KP288321 | KP288322 | KP288323 | KP288324 | KP288325 | KP288326 | KP288327 | KP288328 |
| A/duck/Sichuan/NCXJ15/2014(H5N6) | KM251535 | KM251525 | KM251515 | KM251465 | KM251495 | KM251488 | KM251475 | KM251505 |
| A/duck/Sichuan/NCXN11/2014(H5N1) | KM251540 | KM251530 | KM251520 | KM251470 | KM251500 | KM251483 | KM251480 | KM251510 |
| A/duck/Taizhou/TZYG12/2015(H5N6) | KU143581 | KU143538 | KU143495 | KU143271 | KU143406 | KU143369 | KU143323 | KU143452 |
| A/duck/Vietnam/LBM360c1-4-1/2013(H5N6) | LC010693 | LC010694 | LC010695 | LC010696 | LC010697 | LC010698 | LC010699 | LC010700 |
| A/duck/Vietnam/LBM632/2014(H5N1) | AB979460 | AB979461 | AB979462 | AB979463 | AB979464 | AB979465 | AB979466 | AB979467 |
| A/duck/Vietnam/LBM633/2014(H5N1) | AB979468 | AB979469 | AB979470 | AB979471 | AB979472 | AB979473 | AB979474 | AB979475 |
| A/duck/Vietnam/LBM751/2014(H5N6) | LC028189 | LC028190 | LC028191 | LC028192 | LC028193 | LC028194 | LC028195 | LC028196 |
| A/duck/Wenzhou/YHQL22/2014(H5N6) | KU143580 | KU143537 | KU143494 | KU143269 | KU143405 | KU143367 | KU143322 | KU143450 |
| A/duck/Wuhan/JXYFB22/2015(H5N6) | KU143579 | KU143536 | KU143493 | KU143270 | KU143409 | KU143368 | KU143321 | KU143451 |
| A/duck/Yamagata/061004/2014(H6N6) | LC042078 | LC042079 | LC042080 | LC042081 | LC042082 | LC042083 | LC042084 | LC042085 |
| A/environment/Guangdong/HY243/2015(H5N6) | KT370112 | KT370104 | KT370097 | KT370063 | KT370085 | KT370072 | KT370070 | KT370090 |
| A/environment/Yunnan/YN22/2015(H5N6) | KT963060 | KT963059 | KT963058 | KT963053 | KT963056 | KT963055 | KT963054 | KT963057 |
| A/environment/Yunnan/YN25/2015(H5N6) | KT963068 | KT963067 | KT963066 | KT963061 | KT963064 | KT963063 | KT963062 | KT963065 |
| A/goose/Zhejiang/1120132/2014(H5N6) | KU042676 | KU042705 | KU042734 | KU042763 | KU042792 | KU042821 | KU042850 | KU042879 |
| A/goose/Zhejiang/112080/2014(H5N6) | KU042675 | KU042704 | KU042733 | KU042762 | KU042791 | KU042820 | KU042849 | KU042878 |
| A/goose/Zhejiang/925036/2014(H5N6) | KU042671 | KU042700 | KU042729 | KU042758 | KU042787 | KU042816 | KU042845 | KU042874 |
| A/goose/Zhejiang/925037/2014(H5N8) | KU042682 | KU042711 | KU042740 | KU042769 | KU042798 | KU042827 | KU042856 | KU042885 |
| A/goose/Zhejiang/925105/2014(H5N6) | KU042672 | KU042701 | KU042730 | KU042759 | KU042788 | KU042817 | KU042846 | KU042875 |
| A/goose/Zhejiang/925106/2014(H5N6) | KU042673 | KU042702 | KU042731 | KU042760 | KU042789 | KU042818 | KU042847 | KU042876 |
| A/muscovyduck/QuangNinh/4c111/2013(H5N6) | LC050588 | LC050589 | LC050590 | LC050591 | LC050592 | LC050593 | LC050594 | LC050595 |
| A/pigeon/Sichuan/NCXN29/2014(H5N1) | KM251541 | KM251531 | KM251521 | KM251471 | KM251501 | KM251484 | KM251481 | KM251511 |
| A/swan_goose/Jilin/JL01/2014(H5N6) | KM873643 | KM873644 | KM873645 | KM873646 | KM873647 | KM873648 | KM873649 | KM873650 |
| A/swine/Guangdong/1/2014(H5N6) | KT313409 | KT313408 | KT313407 | KT313418 | KT313405 | KT313404 | KT313403 | KT313406 |
| A/turtledove/Wuhan/WHBJ12/2014(H5N6) | KU143570 | KU143528 | KU143485 | KU143261 | KU143397 | KU143359 | KU143313 | KU143441 |
| A/wild_duck/Shandong/628/2011(H5N1) | JX534562 | JX534563 | JX534564 | JX534565 | JX534566 | JX534567 | JX534568 | JX534569 |
| A/Sichuan/26221/2014(H5N6) | EPI533585 | EPI533586 | EPI533587 | EPI533583 | EPI533588 | EPI533584 | EPI533589 | EPI533590 |
| A/chicken/Vietnam/NCVD-389/2009(H5N1) |  |  |  |  |  |  |  |  |
| A/Duck/Guangdong/S4251/2010(H6N6) | KJ200828 | KJ200827 | KJ200826 | KJ200821 | KJ200824 | KJ200823 | KJ200822 | KJ200825 |
| A/Duck/Guangdong/PY501/2014(H5N6) |  |  |  |  |  |  |  |  |
| A/Pallas's Sandgrouse/Guangdong/ZH283/2015(H5N6) | KT454952 | KT454953 | KT454954 | KT454955 | KT454956 | KT454957 | KT454958 | KT454959 |
| A/Common Moorhen/Guangdong/GZ174/2014(H5N6) | KT454944 | KT454945 | KT454946 | KT454947 | KT454948 | KT454949 | KT454950 | KT454951 |
| A/Oriental Magpie-robin/Guangdong/SW8/2014(H5N6) | KT454936 | KT454937 | KT454938 | KT454939 | KT454940 | KT454941 | KT454942 | KT454943 |
| A/Guangdong/SZ872/2015(H5N6) | EPI_ISL_206568 (Global Initiative on Sharing Avian Influenza Data) | | | | | | | |
| A/Guangdong/ZQ874/2015(H5N6) | EPI_ISL_206569 (Global Initiative on Sharing Avian Influenza Data) | | | | | | | |

Supplementary Table 2. Molecular characterization of the three H5N6 viruses compared with five human-origin H5N6 viruses.

| Viral protein, phenotypic consequences ^†^ | position/motif | SW8 | GZ174 | ZH283 | A/Guangdong/  SZ872/2015 | A/Guangdong/  ZQ874/2015 | A/Guangzhou/39715/2014 | A/Yunan/  0127/2015 | A/Sichuan/  26221/2014 |
| --- | --- | --- | --- | --- | --- | --- | --- | --- | --- |
| **PB2** | | | | | | | | | |
| Mammalian host adaptation | E627K | E | E | E | E | E | K | K | E |
| Cross-speciestransmission in mammals | D701N | D | D | D | D | N | D | D | N |
| **HA** | | | | | | | | | |
| Cleavage site (Required for high pathogenicity) |  | -RRRKR↓G- | -RRRKR↓G- | -RRRKR↓G- | -REKRRKR↓G- | -REKRRKR↓G- | -RRRKR↓G- | -RRRKR↓G- | -KRRKR↓G- |
| Increased virus binding to α-2,6 | S138A | A | A | A | A | A | A | A | A |
|  | I155T | I | T | T | T | T | T | T | I |
| Increased virus binding to α-2,3 (H3 numbering) | Q226L | Q | Q | Q | Q | Q | Q | Q | Q |
|  | G228S | G | G | G | G | G | G | G | G |
| **NA** | | | | | | | | | |
| Stalk 58-68 deletion (Increased virulence in mice) |  | Yes | Yes | Yes | Yes | Yes | Yes | Yes | No |
| Increased sensitivity to oseltamivir | H274Y | H | H | H | H | H | H | H | H |
| **PB1** | | | | | | | | | |
| Enhanced transmission in feretts | I368V | I | I | I | V | I | I | V | I |
| **M1** | | | | | | | | | |
| Increased virulence in mice | N30D | D | D | D | D | D | D | D | D |
|  | T215A | A | A | A | A | A | A | A | A |
| Host marker of influenza A virus | K174R | R | R | R | R | R | R | R | R |
| **M2** | | | | | | | | | |
| Increased sensitivity to amantadine | S31N | S | S | S | N | S | S | N | S |
| **NS1** | | | | |  |  | | | |
| Increased virulence in mammal | P42S | S | S | S | S | S | S | S | S |
|  | D97E | D | D | D | D | E | D | E | E |
|  | I101M | D | D | D | D | D | D | D | D |
| 80-84 deletion (Increased virulence in poultry) |  | Yes | Yes | Yes | Yes | Yes | Yes | Yes | No |
